# Cholera Infantum

**Published:** 1882-08-20

**Authors:** W. F. Hamer


### ON CHOLERA INFANTUM.

BY W. F. HAMER, M. D.

I shall not enter into the general details of this subject, as every practitioner knows what infantile cholera is, but will simply report some cases as they have occurred in my practice.

Case I.—I was called to see M. E., aged eleven months, July 10, 1881, at 10 a. m., and found her vomiting, the bowels acting every ten minutes, the discharges being very watery; pulse 140, temperature 104°. There was considerable stupor. She was placed in a mustard bath from six to ten minutes, and afterward rubbed dry and laid in bed. The following was ordered: Iced gum-water freely as a drink alternately with sub-nit. of bismuth and saccharated pepsin, of each ten grains, given in ice-water every one or two hours. A poultice of mustard and flaxseed was placed over the abdomen and cold applications made to the head. I called at 2 p. m., and found the patient resting easy. The bowels had moved four times and there had been some vomiting; pulse 130, temperature 102½°; treatment continued. I saw her at 7 p. m.; pulse 130, temperature 102°; had vomited two or three times; bowels had acted three times since 1 o'clock. The bath was again resorted to and the following prescription was given:

℞ Tinct. opii deodorat. . . . . . . . . . . . . . . . . . . . . . . . . . gtt. x,

Bismuth subnit. . . . . . . . . . . . . . . . . . . . . . . . . . . . . ʒ ij,

Syrup simpl. . . . . . . . . . . . . . . . . . . . . . . . . . . . . . ℥ ss,

Mist. cretæ. . . . . . . . . . . . . . . . . . . . . . . . . . . . . . . ℥ jss.

Mix. Sig. Teaspoonful every two hours alternately with the gum-water. Iced brandy was also prescribed.

Called at 6 a. m., July 11th: patient resting easy; treatment continued. Called at 11 a. m. There had been some vomiting, but the bowels were easier; pulse 102°. The bath was again given and treatment continued. At 2 p. m. patient was resting well. At 8 p. m. pulse 130, temperature 102½°; bath again given and treatment continued.

At 7 a. m., July 12th, patient had rested well, vomited but twice during the night; the bowels had moved three times; pulse 115, temperature 100°. At 3 p. m., still improving; medicine to be given at longer intervals.

July 13th, at 8 a. m., still improving. Case discharged.

CASE II.—R. H., aged fourteen months. I first saw him on July 14th, at 3 p. m. The bowels were acting frequently and the patient had vomited several times; pulse 120, temperature 103½°; the stools were thin and watery. I ordered the following—

℞ Bismuth subnit.............................. ʒ ijss,

Pulv. cret. camph.............................. c,

Opii.............................. ʒ ss,

Pepsin sacch.............................. ʒ ij.

Mix and divide into ten powders. One powder to be taken every two hours in ice-water alternately with gum-water. A poultice of flaxseed and mustard was applied over the abdomen, moistened with an infusion of hops. Cold applications were made to the head.

At 9 p. m. pulse 130, temperature 104°. A mustard bath was given and iced brandy ordered to be given alternately with the powders.

At 6 a. m., the 15th, the bowels were easier, but the patient had vomited three or four times; pulse 120, temperature 102°. Treatment continued. At 1 p. m. resting at ease. At 7 p. m. pulse 115, temperature 101°; the bowels had moved three times since my last visit; patient had vomited once.

At 8 a. m., July 16th, still improving. Case discharged.

CASE III.—Z. E., aged sixteen months. I visited him on July 17th, and learned from the parents that previous to my call he had had simple diarrhœa for a week or more. At the time of visit the vomiting was persistent, the bowels acting at short intervals; stools very watery and in considerable quantity at each passage; pulse 130, temperature 104°; patient very restless. During this visit the patient was seized with a convulsion, which lasted about twenty minutes. The mustard bath was given and the following prescribed—

℞ Potas. brom.............................. ʒ ij,

Aquæ menth. pip.............................. ℥ ss,

Aquæ destil.............................. ℥ jss.

Mix. A teaspoonful every twenty minutes until quiet is restored. A poultice of mustard and flaxseed was applied over the whole abdomen, and as soon as he became quiet the following prescription was given :

℞ Bismuth subnit ............................ } aa gr. xij,

Pepsin sacch ............................ }

In ice-water, to be repeated every two hours. Cold applications to the head were also ordered.

At 5 p. m. the patient was easy; pulse 120, temperature 102°. Bath again given and treatment continued.

I saw him again at 7 a. m. the 18th. He had vomited some three or four times, and the bowels had moved four times; pulse 115, temperature 101°; treatment continued. At 1 p. m. bowels were acting more frequently and the vomiting continued. The bath was again resorted to and the following was prescribed :

℞ Bismuth subnit.......................... } aa gr. xij

Pepsin sacch .......................... }

Pulv. Dover............................... gr. ss.

Mix. To be given in ice-water every two hours; also iced gum-water alternately. At 8 p. m. the patient was resting well; he had vomited twice and the bowels had acted three times; pulse 115, temperature 101½°. Treatment continued.

At 7 a. m., July 19th, I found that his bowels had moved but three times during the night, and that he had vomited once; pulse 110, temperature 100°; treatment continued. Saw him at 5 p. m., he was still improving, and I discharged the case.

CASE IV.—On August 13th, at 2 o'clock a. m., I was called in haste to see H. R., aged fifteen months. I found him in a violent convulsion, which lasted about thirty minutes; bowels acting very freely, and there was much vomiting. I gave chloroform by inhalation aud had a large mustard poultice applied over the bowels, with smaller ones around the wrists and ankles. The convulsion being under control, he was put upon the following :

℞ Potass. brom................................ ʒ ij,

Aquæ menth. pip............................ ℥ ss,

Aquæ destil................................ ℥ jss.

Mix. A teaspoonful in ice-water every thirty minutes until the patient becomes quiet.

The bismuth and pepsin, as prescribed in the other cases, were given every hour or two in ice-water. Cold applications to the head were also made. At 8 a. m. there was some vomiting, but the bowels were easier. The potass. bromide mixture was ordered to be given every two or three hours with iced gum-water and brandy, and pepsin and bismuth every hour or two.

At 1 p. m. the patient was easy; treatment continued. At 8 p. m. had vomited but twice since my last visit; his bowels had acted four times.

August 14th, 7 a. m., he had rested well during the night; treatment continued. At 5 p. m patient still improving. I ordered the medicine to be given at longer intervals, and on the next day discharged the case.—*Louisville Med. News.*

